# Role of *FTO* rs9939609 and *LEPR* rs1137101 Genetic Variants in Gestational Weight Gain and Neonatal Weight Among Pregnant Adolescents

**DOI:** 10.3390/ijms27083413

**Published:** 2026-04-10

**Authors:** Reyna Sámano, Hugo Martínez-Rojano, Ashley Díaz-Medina, Irma Eloísa Monroy-Muñoz, Gabriela Chico-Barba, María Eugenia Mendoza-Flores, Héctor Borboa-Olivares, Verónica Zaga-Clavellina, Ricardo Gamboa, Melissa Daniela Gonzalez-Fernandez, Ángela Felipe-Hernández, Rosalba Sevilla-Montoya, Alejandro Martínez-Juárez

**Affiliations:** 1 Coordinación de Nutrición y Bioprogramación Instituto Nacional de Perinatología, Mexico City C.P. 11000, Mexico; gabyc3@gmail.com (G.C.-B.); tina14mx@yahoo.com (M.E.M.-F.); 2 Sección de Posgrado e Investigación Escuela Superior de Medicina Instituto Politécnico Nacional, Mexico City C.P. 11340, Mexico; 3 Programa de Maestría en Ciencias de la Salud Escuela Superior de Medicina Instituto Politécnico Nacional, Mexico City C.P. 11340, Mexico; d.ash_a@ymail.com; 4 Departamento de Investigación en Salud Reproductiva Instituto Nacional de Perinatología, Mexico City C.P. 11000, Mexico; irmae4901@gmail.com (I.E.M.-M.); mgonzalezf1001@alumno.ipn.mx (M.D.G.-F.); 5 Subdirección de Investigación Clínica Instituto Nacional de Perinatología, Mexico City C.P. 11000, Mexico; hector.borboa@inper.gob.mx; 6 Dirección de Investigación, Instituto Nacional de Perinatología, Mexico City C.P. 11000, Mexico; veronia.zaga@inper.gob.mx; 7 Departamento de Fisiología Instituto Nacional de Cardiología “Ignacio Chávez”, Mexico City C.P. 14080, Mexico; rgamboaa_2000@yahoo.com; 8 Departamento de Tococirugía y Urgencias Instituto Nacional de Perinatología, Mexico City C.P. 11000, Mexico; anfel64@yahoo.com.mx; 9 Coordinación de Genética y Genomica Humana Instituto Nacional de Perinatología, Mexico City C.P. 11000, Mexico; dra.rosalbasevilla@outlook.com; 10 Departamento de Fisiología y Desarrollo Celular Instituto Nacional de Perinatología, Mexico City C.P. 11000, Mexico; alejandro.martinez@inper.gob.mx

**Keywords:** pregnant adolescents, polymorphisms, genetic variants, gestational weight gain, birth weight, obesity

## Abstract

Gestational weight gain (GWG) and birth weight (BW) have a multifactorial etiology, which makes identifying the most influential determinants difficult. The association between variants of the *FTO* and *LEPR* genes has been explored as contributing factors to obesity in various age groups; however, their role in GWG and BW in adolescent mothers and their offspring is uncertain. To determine whether the presence of polymorphisms rs9939609 (*FTO*) and rs1137101 (*LEPR*) is associated with gestational weight gain and newborn weight in a cohort of adolescent mothers. Methods: A prospective cohort study of 305 mother-child dyads was conducted between 2020 and 2024. Genotyping of the single nucleotide variants (SNVs) rs9939609 of the *FTO* gene and rs1137101 of the *LEPR* gene was performed using real-time PCR and high-resolution melting analysis (qPCR-HRM), using maternal peripheral blood and umbilical cord blood samples. GWG, BW, energy intake, and other perinatal data were recorded and classified. Genetic data from 305 mother–offspring dyads were analyzed. The median maternal age was 16 years, and 71.4% had a normal pre-pregnancy body mass index (BMI). The most frequent genotypes were TT for *FTO* rs9939609 and AG for *LEPR* rs1137101. In both groups, the genotypic distribution significantly deviated from Hardy–Weinberg equilibrium (*p* < 0.0001). The AA genotype of *FTO* was associated with a higher probability of excessive gestational weight gain (GWG) after adjustment for pre-pregnancy BMI and dietary and sociodemographic factors. High protein and lipid intake increased the risk of excessive GWG, whereas adequate intake of carbohydrates and legumes showed a protective effect. An initial significant association was identified between the *LEPR* rs1137101 variant (AA allele) and low birth weight (LBW); however, this association was lost after adjustment for confounding factors. The *FTO* rs9939609 variant was significantly associated with GWG. On the other hand, the *LEPR* rs1137101 variant in the offspring showed an association with BW categorized by percentiles (in crude analysis), while the *FTO* variant showed no relationship with birth weight.

## 1. Introduction

Gestational weight gain (GWG), both excessive and insufficient, has been associated with various long-term adverse outcomes for the mother-child dyad, including hypertensive disorders of pregnancy, gestational diabetes, preterm birth, and large for gestational age (LGA) newborns, among others. Birth weight, reflecting intrauterine conditions, is influenced by multiple factors, including maternal weight, GWG, and dietary intake [1].

During adolescent pregnancy, nutritional needs increase due to metabolic and physiological transformations inherent to both pregnancy and adolescence [2]. The study by Gibbs et al. [3] suggests that a short gynecological age or a very young maternal age at conception or delivery predicts low birth weight, which is related to biological immaturity. This is explained by the potential nutrient competition between the adolescent mother and the fetus, which can lead to adverse outcomes for the infant. Adolescents who are still growing might prioritize their own nutritional requirements over those of the fetus, either due to hormonal changes or insufficient dietary intake. This competition has been associated with placental alterations, glycine deficiency, preterm birth, intrauterine growth restriction, and micronutrient deficiencies, negatively impacting fetal development, particularly in contexts of food insecurity [4].

Maternal obesity and excessive gestational weight gain represent significant risk factors for complications such as gestational diabetes, preeclampsia, and increased fetal adiposity [5]. These modifications are regulated by hormonal processes involving placental growth hormone (PGH), human chorionic somatomammotropin (hCS), ghrelin, leptin, and adiponectin, whose function is likely influenced and mediated by genetic factors [6].

Although obesity is influenced by environmental factors, genetic predisposition also plays a fundamental role in individual susceptibility [7]. This genetic contribution manifests through various mechanisms that control regulatory pathways and systems involved in energy intake and expenditure [8]. Candidate gene studies have identified key genes and single nucleotide polymorphisms (SNPs) that participate in the regulation of eating behavior, including caloric intake, food preferences, and satiety [9]. In particular, relationships have been identified between the rs9939609 SNP of the *FTO* (fat mass and obesity-associated) gene, the rs7799039 SNP of the *LEP* (leptin) gene, and the rs1137101 SNP of the *LEPR* (leptin receptor) gene and total energy intake [10].

In addition to the aforementioned nutritional and metabolic factors, genetic predisposition can also influence food preferences, which in turn impacts long-term food intake and, consequently, weight gain. Furthermore, the *FTO* gene has been shown to function as a key nutritional sensor in the hypothalamus, where it modulates satiety signals and the termination of intake based on nutritional status and amino acid concentrations. When its expression increases, particularly in the arcuate nucleus, food consumption is reduced; in contrast, obesity-linked variants interfere with this satiety signal, promoting increased food intake and altering sensitivity to reward stimuli [11].

In pregnant adult women and their offspring, the rs9939609 (*FTO*) and rs1137101 (*LEPR*) polymorphisms have been studied and shown a positive correlation with birth weight and offspring fat mass index [12]. In various populations, such as the Brazilian one, the rs9939609 (*FTO*) polymorphism has been associated with GWG and has even been shown to be a predictor of postpartum weight gain [1]. Similarly, a genome-wide association study conducted in Poland reported a negative association between the rs1137101 (*LEPR*) polymorphism and GWG in adult women [13].

Regarding the adult Mexican population, only one investigation has reported an association between the rs9939609 (*FTO*) polymorphism and conditions such as threatened abortion and gestational diabetes [14,15,16]. However, there is scarce information on the relationship between these polymorphisms and GWG in Mexican adolescents.

Given the relevance of early identification of overweight and obesity risk factors from birth for long-term health and the crucial influence of maternal weight on birth weight, the detection of single nucleotide polymorphisms (SNPs) associated with the development of obesity and adiposity, highlighting the *FTO* and *LEPR* genes as the most investigated, is of great interest [17]. Therefore, the early detection of these SNPs in newborns is of great interest, with the aim of genotyping the study population and elucidating possible associations between these SNPs, pregestational and gestational maternal body mass index (BMI), and newborn weight.

The objective of the present study was to determine whether the presence of the genetic variants rs9939609 (*FTO*) and rs1137101 (*LEPR*) is associated with gestational weight gain and newborn weight in adolescent mothers.

## 2. Results

The study initially included a sample of 360 participants. Genetic material for the *FTO* rs9939609 variant was available from 353 mothers and 305 children, while for the *LEPR* rs1137101 variant, material was available from 355 mothers and 309 children. In total, 305 mother-child dyads were analyzed for both genetic variants.

The median maternal age was 16 years. The median pre-gestational BMI was 21.6 kg/m^2^, with most participants (71.6%) exhibiting a normal weight status, while a quarter (25.3%) were classified as overweight or obese.

Within this sample, 26.4% of participants achieved adequate gestational weight gain, with a median of 11.7 kg. Low birth weight, defined as less than 2500 g or classified according to Intergrowth standards, was observed in approximately 20% of offspring.

Regarding marital status, three-quarters of the adolescents were single (75.5%). In terms of family structure, 40.5% resided in nuclear families, and only 28.4% of the adolescents cohabited with their partner. Over 90% of participants had a low or very low socioeconomic status. More than half of the participants had secondary education, and a quarter had primary or lower education (Table 1).

### 2.1. Genetic Polymorphism Frequencies

Genotype frequencies for the *FTO* rs9939609 polymorphism showed that 131 mothers were homozygous for the TT genotype, while 224 carried a variant allele (AA or AT). Among neonates, 170 exhibited the TT genotype, and 185 presented variant genotypes. For the *LEPR* rs1137101 polymorphism, 107 mothers displayed the homozygous AA genotype, and 248 carried a variant allele (GG or AG). In neonates, 116 were homozygous for the AA genotype, with 239 carrying variant genotypes.

**Figure 1:**
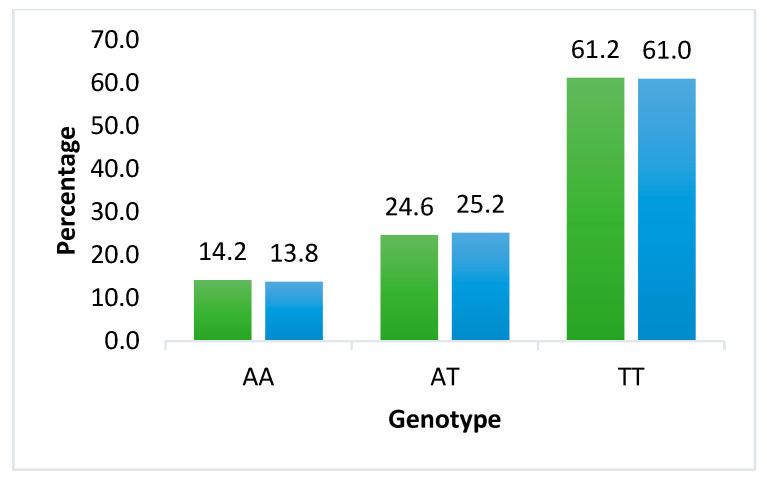
*FTO* rs9939609 genotype frequencies in mothers (green bars) and offspring (blue bars).

**Figure 2:**
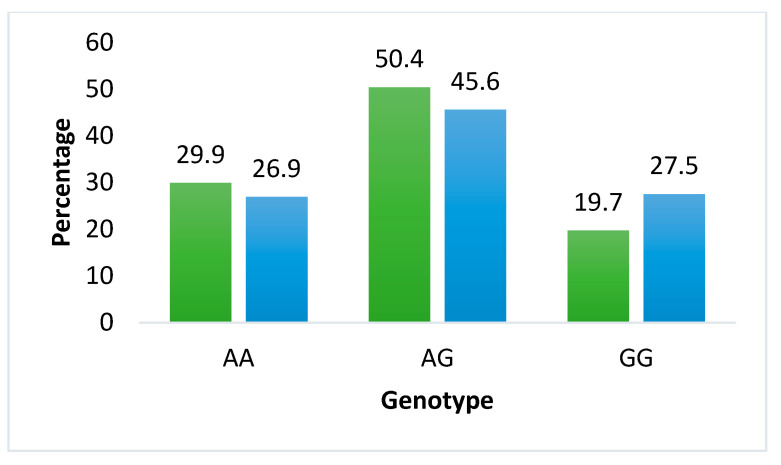
*LEPR* rs1137101 genotype frequencies in mothers (green bars) and offspring (blue bars).

Overall, the TT genotype of *FTO* rs9939609 was the most prevalent in both mothers and neonates. In contrast, for *LEPR* rs1137101, the heterozygous AG genotype was the most common among the variant genotypes observed in both groups (Figure 1 and Figure 2).

For the *FTO* rs9939609 polymorphism, the homozygous TT genotype was the most prevalent in both mothers (*n* = 353; 61.2%) and offspring (*n* = 305; 61.0%). This was followed by the heterozygous AT genotype and, lastly, by the homozygous AA genotype (representing the risk allele). In both groups, the genotypic distribution significantly deviated from Hardy–Weinberg equilibrium (*p* < 0.0001).

Similarly, for the *LEPR* rs1137101 polymorphism, the heterozygous AG genotype was the most frequent in both mothers (50.4%) and offspring (45.6%), followed by the homozygous AA and then the GG genotypes. Unlike the *FTO* polymorphism, the distribution of this variant conformed to Hardy–Weinberg equilibrium in both mothers (*p* = 0.720) and offspring (*p* = 0.120) (see Supplementary Table S2).

Regarding gestational weight gain (GWG), no significant differences were observed in its distribution across *FTO* rs9939609 genotypes in adolescent mothers (*p* > 0.05). Likewise, when analyzing GWG in relation to *LEPR* rs1137101 genotypes, no significant differences were found (*p* = 0.917).

Comparison of genotypic distributions between generations using the chi-square test for homogeneity revealed statistically significant differences for both polymorphisms (*FTO* rs9939609: χ^2^ = 20.0, *p* < 0.001; *LEPR* rs1137101: χ^2^ = 33.04, *p* < 0.001) (see Table 2).

Maternal genotypes of the *FTO* rs9939609 and *LEPR* rs1137101 polymorphisms were not significantly associated with birth weight (*p* > 0.05). Similarly, no association was observed between offspring *FTO* rs9939609 genotypes and their birth weight, whether expressed as Z-scores or percentiles (*p* > 0.05).

However, for the *LEPR* rs1137101 polymorphism, a specific analysis stratified by percentiles revealed a significant difference: neonates carrying the heterozygous genotype exhibited significantly higher birth weight, both when expressed as percentiles and absolute values (*p* = 0.048) (see Table 3).

Analysis of nutrient and food intake distribution relative to the *FTO* rs9939609 and *LEPR* rs1137101 genotypes revealed no statistically significant differences. The consumption of carbohydrates, lipids, and proteins was comparable across all genotypes. Additionally, a generally low intake of legumes was noted (Supplementary Table S3).

### 2.2. Associations with Gestational Weight Gain and Birth Weight

In the adjusted models (models 3 and 4), the homozygous AA variant of the *FTO* rs9939609 polymorphism was associated with a significantly higher probability of excessive gestational weight gain (GWG) (OR: 4.350). These models were adjusted for high nutrient intake, pre-gestational BMI (classified as overweight/obesity), and sociodemographic variables. It is important to note that high protein and lipid intake, low legume consumption, and elevated pre-gestational BMI were also identified as factors increasing the risk of excessive GWG. Conversely, in the unadjusted models (model 1), no significant association was observed between maternal genetic variants and either excessive or insufficient GWG. For insufficient GWG, no maternal genetic variant showed a significant association; only high protein intake was found to be related to this outcome (Table 4).

In the case of the association of the *FTO* rs9939609 and *LEPR* rs1137101 polymorphisms and other variables with excessive and insufficient gestational weight gain, no statistically significant gene-diet interactions were identified for any of the outcomes. The interactions between *FTO* rs9939609 and *LEPR* rs1137101 and the intake of protein, lipids, or legumes did not reach statistical significance in the logistic regression models (*p* > 0.15 in all cases). Although protein intake independently contributed to the risk of both excessive and insufficient gestational weight gain, its effect, as well as that of lipid or legume intake, was not observed to be modified by the maternal *FTO* or *LEPR* genotypes (Table 4).

Regarding low birth weight (<2500 g), maternal and offspring genetic variants of *FTO* rs9939609 and *LEPR* rs1137101 were not associated with its presence in any of the four adjusted models. Only high maternal carbohydrate and lipid intake demonstrated a relationship with low birth weight (Table 5).

Regarding low birth weight (<2500 g), and after adjusting for nutritional, anthropometric, and sociodemographic factors, no statistically significant gene-diet interactions were identified between the maternal or offspring genetic variants *FTO* rs9939609 and *LEPR* rs1137101 and any of the dietary factors investigated. The interaction terms involving *FTO* rs9939609 or *LEPR* rs1137101 with lipid and carbohydrate intake did not reach statistical significance in the logistic regression models (*p* > 0.23 in all cases). The dietary factors independently associated with low birth weight were not modified by maternal or offspring genotypes (Table 5).

## 3. Discussion

In the current study, the homozygous AA genotype of the *FTO* rs9939609 polymorphism was found to be associated with a higher probability of excessive gestational weight gain (GWG) after adjustment for pre-gestational BMI, dietary, and sociodemographic variables. High protein and lipid consumption also increased the risk of excessive GWG, while low legume intake was similarly associated with an elevated risk.

An initial significant association was identified between the *LEPR* rs1137101 genetic variant (AA genotype) and low birth weight. However, this association was no longer significant after adjustment for factors such as pre-gestational BMI, dietary, and sociodemographic variables.

The *FTO* rs9939609 allelic frequencies observed in our research are consistent with those reported in international databases. Specifically, the National Center for Biotechnology Information (NCBI) Allele Frequency Aggregator (ALFA) [18] describes frequencies of A = 0.253 and T = 0.747 for the “Latinoamérica 2” population. This population is characterized as mestizo, with predominantly European and Native American ancestry. In our INPer cohort of adolescent mothers, the allelic frequencies were A = 0.264 and T = 0.735, while for the offspring, values of A = 0.263 and T = 0.736 were found; both sets of values are very similar to those reported. Notably, in both groups (mothers and offspring), the genotypic distribution of *FTO* rs9939609 significantly deviated from Hardy–Weinberg equilibrium (HWE), a finding consistent with observations in other populations. This concordance with Mexican mestizo populations is relevant, given that previous studies in Mexico have documented variability in the distribution of *FTO* rs9939609 alleles, showing a higher prevalence of the A allele in individuals with European ancestry and the T allele in those with Native ancestry [19].

Regarding the *LEPR* rs1137101 polymorphism, NCBI/ALFA reported allelic frequencies of A = 0.5440 and G = 0.4596 for the “Latinoamérica 2” population [20]. In our INPer cohort, similar frequencies were obtained for mothers (A = 0.5507, G = 0.4492) and for offspring (A = 0.4967, G = 0.5032), with the genotypic distribution conforming to HWE in both groups.

While the genotypic distribution of the *LEPR* rs1137101 polymorphism conformed to HWE, this was not the case for *FTO* rs9939609, whose distribution significantly deviated, consistent with previously reported variability in Mexico [19]. It should be noted that, in our study population, a similar allelic proportion was maintained across the two generations analyzed (mothers and offspring), suggesting consistency in the genetic composition of both generations within the cohort.

The observed deviation from Hardy–Weinberg equilibrium (HWE) in our cohort could be attributed to several reasons:

Specific inclusion criteria: The study cohort is not a random sample of the general population; rather, it consists of adolescent mothers recruited under specific criteria (age, prenatal care at a single hospital, and possible over-representation of certain BMIs or complications) [21].

Genotype-phenotype association: If the *FTO* polymorphism is strongly associated with traits such as obesity or eating behavior, which in turn can influence inclusion/exclusion criteria or the phenotype of interest, the genotype frequency in the sample could be distorted relative to the reference population, resulting in an HWE deviation. This is particularly relevant if there is an over-representation of a specific phenotype (e.g., excessive gestational weight gain) linked to the risk allele [22].

Population stratification: Mexico (and, in general, Latin America) presents a mixture of ancestries (European, indigenous, African) and, frequently, subpopulations with different allele frequencies. If the adolescents come from distinct neighborhoods, municipalities, or indigenous groups with differentiated ancestries, the mixing of these subgroups can generate an HWE deviation [23].

Study design effects: When a locus is strongly associated with the trait that defines the cohort (for example, if cases with excessive gestational weight gain or obesity had been enriched), the sample ceases to behave as an ideal Mendelian population. In such a scenario, the HWE test may reject equilibrium precisely due to that genotype-phenotype association, exacerbated by the inherent selection in the study design [24].

Random fluctuation (sample size): With a sample size of *n* = 305, the probability of a random deviation is lower, though not impossible, especially if the allele frequency is extreme or if subgroups are analyzed. However, the *p*-value < 0.0001 suggests that the deviation is marked and hardly attributable solely to chance [25].

Regarding methodology, genotyping errors were, as far as possible, ruled out through rigorous quality controls (including internal controls, repetitions, and call rate evaluation).

Finally, this HWE deviation represents a limitation of the study that could influence the generalizability of the results; nevertheless, it does not necessarily invalidate the observed association, given that the robustness of the genotyping was ensured.

In our study, the AA genotype of *FTO* rs9939609 was the only genetic factor significantly associated with excessive gestational weight gain, which is consistent with previous evidence suggesting that *FTO* risk variants can increase susceptibility to greater energy storage. Mechanistic data indicate that these variants can modulate the expression of downstream target genes, particularly *IRX3* and *IRX5*, thus promoting white adipocyte differentiation and increasing lipogenic capacity [26,27]. This mechanism offers a biologically coherent framework for interpreting our findings. Notably, in this adolescent population, environmental exposures exerted a more pronounced influence: a higher intake of proteins and lipids, a low consumption of legumes, and an elevated pre-pregnancy BMI were the strongest predictors of excessive gestational weight gain. Taken together, these results underscore the dominant role of modifiable dietary and metabolic factors over genetic variants in this context.

Gestational weight gain (GWG) was adequate in only one-quarter of our study participants. Specifically, 38% presented with insufficient GWG and 36% with excessive GWG. This pattern contrasts with global data, where 53% have been reported with low GWG and 22% with excessive GWG [28]. On the other hand, the mean GWG in our participants was 11.7 kg, which is comparable to the mean reported at the national level [29].

In this study, regression analysis revealed a significant association between the homozygous AA genotype of the *FTO* rs9939609 polymorphism and an increased risk of excessive gestational weight gain (EGWG) in adolescent mothers. This association, which became more pronounced and maintained its statistical significance even after adjusting for dietary variables and pregestational BMI, suggests an important role for *FTO* rs9939609 in the predisposition to EGWG. Additionally, the results highlighted that adolescents who are overweight or obese before pregnancy are almost ten times more likely to experience EGWG.

The study found that a high intake of protein and lipids was associated with an increased risk of excessive gestational weight gain (EGWG), while an adequate intake of carbohydrates and a varied diet had a protective effect. The impact of sociodemographic variables was limited, possibly due to the homogeneity of the sample (mostly from low socioeconomic strata), which may have reduced the ability to detect differences in this factor.

Several recent studies and reviews have described the relationship between *FTO* rs9939609 variants and body mass index, adiposity, and eating behaviors that predispose to weight gain in diverse populations, including children and adolescents [30,31,32]. Recent research also suggests that these variants, including the *FTO* polymorphism, can influence the predisposition to excessive gestational weight gain during pregnancy, possibly through mechanisms of appetite, diet composition, and insulin sensitivity [33,34,35]. However, logistic regression models did not show statistically significant associations between the genetic variants of the *FTO* rs9939609 polymorphism and insufficient gestational weight gain.

Our findings are consistent with evidence from studies in adult populations, showing that the genetic association of the *FTO* gene is clearly manifested when dietary factors and maternal nutritional status are considered together, suggesting a relevant gene-environment interaction during gestation [33,36]. Taken together, the results indicate that the most relevant determinants of excessive gestational weight gain in this population were dietary factors and the mother’s nutritional status before pregnancy.

It is important to note that the scientific literature on the *FTO* rs9939609 variant and gestational weight gain is not entirely homogeneous. Some studies in adult women have not found a significant effect or only mild effects in specific subgroups (e.g., with pre-existing obesity or insulin resistance) [1,14,37]. These discrepancies could be due to differences in the genetic composition of populations (allelic frequency and ancestry), study design (adolescents versus adults), sample size, and covariate control. Therefore, it is suggested that results be interpreted with caution, especially in samples with distinct ancestral compositions.

The persistence of the effect of the *FTO* rs9939609 AA genotype across the different models suggests that this variant could modulate maternal susceptibility to excessive gestational weight gain, especially in the presence of an unbalanced diet and pre-pregnancy overweight/obesity. Collectively, the results from the four models demonstrate that excessive gestational weight gain has a multifactorial nature, involving both genetic components (such as the *FTO* rs9939609 variants) and nutritional and anthropometric factors. This underscores the importance of the nutritional environment and its interaction with genetic predisposition in modulating maternal weight during gestation.

In our models, the *LEPR* rs1137101 polymorphism did not show a significant association with gestational weight gain (excessive or insufficient) nor with low birth weight. The existing literature on *LEPR* is heterogeneous; while some series and meta-analyses link its variants to adiposity, leptin levels, or elevated BMI, other research reports weak or no effects [6]. Our results are consistent with studies conducted in India and Mexico, where no effects of this variant on GWG or birth weight were observed [14,38]. However, an association has been documented in a Romanian population between *LEPR* rs1137101 and neonatal weight, where maternal fat mass index influenced the BMI of newborns carrying the G allele [12].

Regarding birth weight and its classification (<2500 g), we found no significant differences associated with the maternal genotype of *FTO* rs9939609 or *LEPR* rs1137101. In the offspring, the only significant association emerged in the analysis by percentiles for *LEPR* rs1137101, where AA homozygotes showed a greater predisposition to being small for gestational age, coinciding with previous reports in a northern Mexican population [14].

Our data support the evidence of modest or absent effects of these variants on birth weight. It is postulated that these variants act indirectly through the maternal phenotype (BMI, appetite, GWG) or that their expression is conditioned by interactions with the intrauterine environment (glycemia, maternal diet) [39,40]. The lack of association with *FTO* aligns with other literature [41], suggesting that its effect is too subtle to be detected during gestation, a period characterized by robust homeostatic growth mechanisms. It is likely that the influence of these genes on weight manifests more clearly in postnatal stages [42].

Physiopathologically, the *FTO* gene encodes a nucleic acid demethylase involved in energy homeostasis and appetite regulation via hypothalamic pathways, favoring higher caloric intake and weight gain in obesogenic environments [43]. During gestation, it is plausible that these mechanisms are modulated by the hormonal and metabolic environment, generating genotype-dependent weight gain trajectories.

Although the *LEPR* gene is crucial for energy metabolism control and is associated with obesity risk [44], its complexity and environmental variability could mask its direct effects on gestational weight gain or birth weight through physiological compensatory mechanisms [45]. On the other hand, the gene-diet interaction detected with *FTO* supports nutrigenetic modulation. Given this multifactorial complexity, the use of genetic panels or polygenic risk scores is suggested as a more robust tool to comprehensively estimate risk [46].

In contrast to previous findings [47], this study did not find an association between macronutrient intake or lipid profile and genetic variants. It is postulated that this discrepancy could be due to gene-diet interaction, given that the effect of these variants is susceptible to dietary modulation (e.g., *FTO* rs9939609 is attenuated with low-fat diets [48]; the association of *LEPR* rs1137101 with obesity risk can be modified by dietary interventions [49]). This suggests that the lack of a direct association in this study could be due to modulation by the specific dietary pattern of its population, which is conditioned by various environmental and socioeconomic factors.

The influence of pregestational BMI and inadequate macronutrient intake on excessive gestational weight gain (EGWG) is confirmed. On the other hand, insufficient GWG was not associated with maternal genetic variants, but it was associated with diet: a high protein intake showed a protective effect against insufficient GWG, which aligns with the literature [50,51,52]. These results highlight the importance for public health of prioritizing nutritional and weight control in adolescent prenatal care, with early identification of these factors being key to optimizing preventive strategies [25].

No significant gene-diet interactions were identified for any outcome. While the intake of protein, lipids, and legumes was independently associated with gestational weight gain or low birth weight, these effects were not modified by maternal or offspring *FTO* or *LEPR* genotypes, suggesting that dietary influences operated largely independently of the variants evaluated. This is consistent with previous evidence indicating that interactions involving common SNPs can be modest and require larger samples or more extreme dietary exposures for their detection.

### 3.1. Limitations

One of the main limitations of the study is the sample size, which, although reasonable, could have limited the statistical power to detect modest genetic associations or complex interactions, especially in stratified analyses. In this regard, the deviation from Hardy–Weinberg equilibrium observed in the *FTO* gene suggests a possible population substructure. These two combined circumstances demand that the results be interpreted with caution and underscore the need for replication in larger, independent cohorts [19]. Future studies with broader cohorts should also consider applying corrections for multiple comparisons when evaluating a more extensive set of interactions.

Regarding dietary variables, although validated instruments were used, the data are susceptible to recall and classification biases. On the other hand, given that the study population consists of adolescents attending a public institution, characteristics of social vulnerability predominate. The high prevalence of unplanned pregnancies and the frequent lack of social support in this group can influence both diet quality and adherence to prenatal care, which introduces confounding variables intrinsic to this population and difficult to control.

Finally, the cross-sectional and observational nature of the design prevents establishing causality; therefore, these associations must be confirmed through experimental and longitudinal studies to elucidate the underlying mechanisms.

### 3.2. Perspectives

Our results suggest that, while genetics (*FTO*) may play a role in the predisposition to inadequate gestational weight gain in adolescents, dietary factors and pregestational BMI remain the most immediate targets for interventions. However, the genetic findings open up a significant avenue for future research to better understand the pathophysiology and enable more personalized medicine in adolescent pregnancy.

## 4. Materials and Methods

The study encompassed a prospective cohort of pregnant adolescents receiving prenatal care at the National Institute of Perinatology (INPer) “Isidro Espinosa de los Reyes” in Mexico City. Participant recruitment occurred between January 2021 and December 2023, with follow-up extending until December 2024.

### 4.1. Participant Recruitment

All pregnant adolescents aged 10 to 19 years who attended the INPer outpatient clinic were invited to participate. Of the 450 invited adolescents, 377 were recruited through consecutive non-probability sampling and agreed to participate; however, only 360 of them met the selection criteria and had complete data for the variables of interest. Written informed consent was obtained from parents or legal guardians, and informed assent was obtained from the adolescents. These procedures were conducted in accordance with the study protocol approved by the Ethics and Biosafety Committee of the National Institute of Perinatology Isidro Espinosa de los Reyes, Ministry of Health (Registration number: INPer-2017-2-101).

### 4.2. Participant Selection Criteria

Inclusion criteria comprised pregnant adolescents who were primigravida, had no history of infectious diseases, and expressed the intention to continue prenatal care and delivery at INPer. Adolescents were excluded if their pregnancies resulted from rape or if they reported alcohol and/or toxic substance use, adherence to vegetarian or vegan diets, eating disorders, or a history of chronic or degenerative diseases. Participants were withdrawn from the study if they withdrew before delivery, revoked informed consent, or if congenital defects were detected in the newborn.

### 4.3. Data Collection

Upon providing informed consent, adolescents who met the selection criteria were scheduled for data collection. Between 30 and 36 weeks of gestation, a standardized questionnaire was administered to obtain data on the following maternal variables: age (years), pre-pregnancy weight (kg), height (m), educational attainment (years), occupation, socioeconomic status (according to the categories of the Mexican Association of Market Research and Public Opinion Agencies A.C.) [53], dietary intake, and hours of sleep. Gestational age was determined by the first ultrasound (performed before 24 weeks of gestation).

### 4.4. Adolescent Anthropometric Parameters

Anthropometric measurements were taken by a physician and two trained nutritionists. Weight (kg) was measured using an InBody 770^®^ Body Composition Analyzer (InBody Co., Ltd., Seoul, Korea), and height (m) was measured with a SECA 222 scale (Hamburg, Germany, 0.1 cm accuracy). Final weight and height measurements, used for gestational weight gain calculation, were taken one to two weeks prior to delivery. In cases of preterm birth, this information was extracted from medical and nursing notes at the time of the woman’s admission for labor. Pre-pregnancy weight was obtained through direct questioning of the participant; the literature has demonstrated the reliability of self-reported pre-pregnancy weight [54]. Pre-pregnancy BMI was calculated and classified according to the 2006 World Health Organization (WHO) guidelines for adolescents [55].

### 4.5. Calculation of Gestational Weight Gain and Percentage of Adequacy

Gestational weight gain (GWG) was calculated as the difference between pre-pregnancy weight and the last weight recorded one or two weeks before delivery. In cases of preterm birth, this information was extracted from medical and nursing notes at the time of the woman’s admission for labor. To determine the percentage of adequacy, recommended GWG was calculated based on pre-pregnancy BMI, using WHO percentiles 2006 [55] and following the recommendations of the US Institute of Medicine (IOM). The ratio between actual GWG and recommended weight gain was then calculated, and the result was classified as <90% (Insufficient), 90–125% (Adequate), and >125% (Excessive).

### 4.6. Neonatal Variables

Information was collected on the newborn’s sex, gestational age, weight, length, and head circumference, as well as the number of prenatal care visits and the mode of delivery. Birth weight and length were measured within the first hour post-delivery using precision equipment (SECA 374, “Baby and Mommy” model; precision 0.1 g, and SECA 416 stadiometer; precision 0.1 cm). Low birth weight was defined as less than 2500 g [4]. Birth weight classification followed the Intergrowth criteria, defining categories as SGA (small for gestational age, percentile < 10), AGA (appropriate for gestational age, percentile 10–90), and LGA (large for gestational age, percentile > 90) [56].

### 4.7. Assessment of Dietary Intake

Dietary consumption was assessed using a multi-pass 24 h recall, with data collected between 30 and 36 weeks of gestation. The interview followed an open format, recording detailed information on each food consumed, including type, quantity, preparation method, and time of consumption. To facilitate accurate estimation of portion sizes and reduce potential bias, participants used food replicas, cups, and spoons during the interview, starting with the first food consumed in the morning and continuing chronologically until the last one ingested before bed. The 24 h recalls (two weekdays and one weekend day) were then analyzed using NutriKcal(R) software (NutriKcal^®^ software https://www.nutrikcal.mx/NutriKcalVO.html (accessed on 8 January 2026) (Mexico City, Mexico)) to determine energy and macronutrient intake (carbohydrates, lipids, and proteins) in grams and kilocalories. High intake of nutrients or food was defined as consumption exceeding 120% of the recommended daily allowance for age and physiological state. Correspondingly, low intake was defined as <80% of the recommendation, while adequate intake ranged from 80 to 120%.

### 4.8. Sample Collection for Genotyping

Two types of biological samples were collected: maternal peripheral blood, obtained during the first prenatal visit (between 30 and 36 weeks of gestation)—a period during which all baseline information was also gathered—; and a 5 cm segment of umbilical cord, derived from the placental remnant at birth. From the umbilical cord, 120 mg of blood vessels were dissected for endothelial cell extraction. For maternal blood, leukocytes were isolated by centrifugation.

### 4.9. Obtaining Genetic Material (DNA)

Genomic DNA was extracted from maternal peripheral blood samples and offspring umbilical cord samples. DNA extraction was performed using the commercial Wizard^®^ Genomic DNA Purification Kit (Promega Corporation, Madison, WI, USA). DNA samples were quantified in duplicate by spectrophotometry using a NanoDrop™ 2000 (Thermo Fisher Scientific™, Waltham, MA, USA) and stored at −20 °C until use.

### 4.10. Determination of Genetic Variants

Genetic variants *FTO* rs9939609 and *LEPR* rs1137101 were determined by quantitative real-time PCR (qPCR) coupled with high-resolution melting (HRM) analysis. All experiments were performed in duplicate.

Genotyping reactions were prepared using the Precision Melt Supermix™ kit (Bio-Rad, Hercules, CA, USA), which contains the SYBR Green fluorescent dye. Each reaction contained 1 µL of genomic DNA (adjusted to 30 ng/µL) and specific primers at a final concentration of 10 nM.

The primer sequences used were as follows (also detailed in Supplementary Table S1):

*FTO* fw: 5′-CAGTTATGCATTTAGAATGTCTG-3′

*FTO* rv: 5′-GCTCTCCCACTCCATTTCTGA-3′

*LEPR* fw: 5′-AACAGCCAAACTCAACGACAC-3′

*LEPR* rv: 5′-TTTAGACCTATTATCATCATTT-3′

qPCR and HRM analyses were performed using the CFX96 Touch real-time PCR detection system (Bio-Rad, Hercules, CA, USA). PCR cycling conditions included an initial denaturation at 95 °C for 2 min, followed by 34 cycles. Each cycle consisted of denaturation at 95 °C for 10 s, annealing for 30 s (at 52 °C for *FTO* and 55.7 °C for *LEPR*), and extension at 72 °C for 30 s. The PCR protocol concluded with a final extension at 72 °C for 2 min.

Following PCR, HRM analysis was performed. This consisted of denaturation at 95 °C for 30 s and annealing at 65 °C for 30 s. Product fluorescence was continuously measured from 65 °C to 95 °C, in 0.1 °C increments every 10 s. HRM curves were analyzed using Precision Melt Analysis™ v.1.3 software (Bio-Rad). Genotypes were assigned by comparison with previously genotyped controls [57].

### 4.11. Ethical Considerations

The present study was conducted in strict compliance with the ethical principles established in the Declaration of Helsinki for research involving human subjects. The research protocol was reviewed and approved by the Research, Ethics, and Biosafety Committees of the National Institute of Perinatology “Isidro Espinosa Reyes”, Ministry of Health of Mexico (Registration number: INPer-2017-2-101; approval date: 10 April 2019). Participation was voluntary, and free and informed written consent was obtained from all participants. For adolescent mothers, written informed assent was obtained from the participant, and informed consent was secured from their legal guardians, in the presence of two witnesses who certified the process. Invitations to participate were made in a private environment within the obstetrics service of INPer, guaranteeing the absence of coercion.

### 4.12. Statistical Analysis

Maternal characteristics were summarized using frequencies and percentages for categorical variables, and medians [interquartile ranges (IQR), 25th–75th percentiles] or means (with 95% confidence intervals, CIs) for continuous variables, as appropriate. Normality of distributions was assessed using the Shapiro–Wilk test. Anthropometric variables were expressed as mean ± standard deviation (SD).

The distributions of the studied polymorphisms were described using absolute and relative frequencies. Preliminary associations between the *FTO* rs9939609 and *LEPR* rs1137101 genetic variants (in mothers and offspring) with gestational weight gain were analyzed using the chi-square test. Comparison of means or medians was performed based on data distribution. Additionally, Pearson’s chi-square test was used to assess Hardy–Weinberg equilibrium (see Supplementary Table S2).

For genetic analyses, bivariate associations were evaluated under a codominant model, where each genotype category was analyzed separately. For multivariate regression models, we used an additive inheritance model, coding genotypes as 0 (reference), 1 (heterozygous), and 2 (homozygous variant). Binary logistic regression models were constructed to identify associations between genotypic variants and inadequate gestational weight gain (excessive or insufficient).

Gene-diet interactions were evaluated by adding multiplicative interaction terms to the logistic regression models. Specifically, interaction terms were created for each genetic variant (*FTO* rs9939609 and *LEPR* rs1137101) with dietary exposures previously identified as having independent associations with the outcomes of interest (protein intake, lipid intake, and legume intake). Interaction terms (genotype × nutrient) were introduced into separate models for excessive and insufficient gestational weight gain. All models were adjusted for relevant maternal covariates.

The statistical significance level for all analyses was set at *p* < 0.05 (two-tailed). Statistical analyses were performed using SPSS version 23 (IBM Inc., Armonk, NY, USA).

## 5. Conclusions

In this cohort of adolescent mothers, the homozygous AA genotype of the *FTO* rs9939609 polymorphism was significantly associated with a higher risk of excessive gestational weight gain (GWG), even after adjustment for dietary intake and pre-pregnancy BMI. Conversely, the *LEPR* rs1137101 polymorphism demonstrated no significant association with maternal GWG. Regarding birth weight, the *LEPR* rs1137101 variant in the offspring showed an initial significant association with low birth weight in crude analyses; however, this association was lost upon adjustment for confounding factors. No significant effects on birth weight were observed for the *FTO* rs9939609 variant (in mothers or offspring) or for maternal *LEPR* rs1137101.

Our findings underscore that the most robust determinants of inadequate GWG were dietary factors and maternal pre-pregnancy nutritional status, suggesting a complex interplay between genetic predisposition and environmental influences. From a public health perspective, these results emphasize the critical importance of early nutritional counseling and weight management strategies during prenatal care for adolescent mothers.

Nevertheless, acknowledging the study’s limitations—particularly the observed deviation from Hardy–Weinberg equilibrium for *FTO* rs9939609 and the limited statistical power—these findings should be interpreted with caution as preliminary results. Further replication in larger independent cohorts with appropriate control for population structure and ancestry is essential to confirm these associations and elucidate the underlying mechanisms.

## Figures and Tables

**Table 1:** Clinical and sociodemographic characteristics of the participants.

Characteristics	*n* = 355	
	Median (P25, P75)	Minimum-Maximum Value
Age (years)	16 (15, 17)	11–19
Pre-gestational weight (kg)	52 (47, 59)	35–97
Height (cm) ^a^	155.7 ± 5.8	141–170
Pre-gestational BMI (kg/m^2^)	21.6 (19.8–23.8)	15.0–35.6
Body Mass Index ^b^	Underweight	11 (3.4)
	Normal	257 (71.4)
	Overweight	52 (15.3)
	Obese	35 (10.0)
Pregnancy outcome ^b^	Cesarean section	166 (46.6)
	Vaginal delivery	189 (53.4)
Gestational weight gain ^b^	Insufficient	133 (37.0)
	Adequate	94 (26.4)
	Excessive	128 (36.7)
GWG (kg)	11.7 (8–15.5)	−5–46
GWG adequacy (%)	10.4 (73–154)	−71–488
Birth weight (g)	2894 (2642–3190)	1195–4115
	Low birth weight (<2500 g)	69 (19.4)
	Small for gestational age (<p10, by Intergrowth)	79 (22.1)
Length (cm) ^a^	48.3 ± 2.7	32.5–54
Gestational age (Capurro)	38.5 (37.4–39.4)	23–42
Sociodemographic Characteristics		
Marital status ^b^	Single	268 (75.5)
	Consensual union	87 (24.4)
Family type ^b^	Nuclear	143 (40.5)
	Extended	41 (11.5)
	Compound	22 (6.3)
	Single parent	52 (14.9)
	Did not respond	97 (26.8)
Religion ^b^	Catholic	190 (54.6)
	Christian	26 (7.5)
	None	97 (27.9)
Socioeconomic level ^b^	Very low	145 (40.8)
	Low	184 (51.9)
	Middle	26 (7.3)
Education level ^b^	Primary or less	91 (25.6)
	Secondary	211 (59.2)
	High school	53 (15.2)

The data are presented as follows: (^a^) mean ± standard deviation and (^b^) frequency (%), as applicable. Abbreviations: BMI: Body Mass Index; GWG: Gestational Weight Gain.

**Table 2:** Genotypic frequencies of *FTO* rs9939609 and *LEPR* rs1137101 polymorphisms in mothers and offspring.

*FTO* rs9939609				
Offspring	Mother AA	Mother AT	Mother TT	p *
AA	13 (31.7)	6 (8.5)	23 (12.1)	<0.001
AT	9 (22)	27 (38)	41 (21.6)	
TT	19 (46.3)	38 (53.5)	126 (66.3)	
*LEPR* rs1137101				
Offspring	Mother AA	Mother AG	Mother GG	p *
AA	40 (43)	37 (24.5)	6 (9.7)	<0.001
AG	43 (46.2)	68 (45)	28 (45.2)	
GG	10 (10.8)	46 (30.5)	28 (45.2)	

Data are presented as frequency (%); * *p*-values were calculated using Pearson’s χ^2^ test.

**Table 3:** Birth weight according to *FTO* rs9939609 and *LEPR* rs1137101 genotypes in offspring.

*FTO* rs9939609				
**Variable**	**AA**	**AT**	**TT**	***p***
Birth weight (g) ^a^	3052 (2745–3190)	2920 (2600–3240)	2880 (2650–3195)	0.500
Birth weight (Z-score)	−0.59 (−1.03, −0.21)	−0.74 (−1.21, −0.07)	−0.75 (−1.24, 0.04)	0.235
Birth weight (Percentile)	30.1 (17.1, 47.8)	24.9 (11.6, 51.9)	22.7 (11.2, 51.5)	0.233
Birth weight categories				
Low birth weight ^b^	1 (3.8)	6 (23.1)	19 (73.1)	0.181
Adequate birth weight	40 (14.4)	70 (25.3)	167 (60.3)	
Excessive birth weight	1 (50)	1 (50)	0 (0)	
*LEPR* rs1137101				
Birth weight (g) ^a^	2870 (2589–3122)	2976 (2630–3275)	2875 (2674–3160)	0.345
Birth weight (Z-score)	−0.97 (−1.23, −0.26)	−0.62 (−1.14, 0.12)	0.71 (−1.22, −0.17)	0.233
Birth weight (Percentile)	17.4 (11.3, 39.5)	31.5 (14, 56.4)	24.2 (12.8, 24.3)	0.048
Birth weight categories				
Low birth weight ^c^	7 (26.9)	12 (46.2)	7 (26.9)	0.661
Adequate birth weight	76 (27)	127 (45.2)	78 (27.8)	
Excessive birth weight	0 (0)	2 (100)	0 (0)	

(^a^) Data are presented as median (interquartile range: P25, P75) for continuous variables; *p*-values were calculated using the Kruskal–Wallis test. (^b^) Data are presented as n (%) for categorical variables; *p*-values were calculated using Pearson’s χ^2^ test. (^c^) Data are presented as *n* (%) for categorical variables; *p*-values were calculated using Fisher’s exact test.

**Table 4:** Association of *FTO* rs9939609 and *LEPR* rs1137101 polymorphisms and other variables with excessive and insufficient gestational weight gain.

Variable	Excessive GWG			Insufficient GWG		
	OR	IC 95%	*p*	OR	IC 95%	*p*
Model 1						
*FTO* rs9939609 (AA)	2.278	0.965–5.378	0.060	1.043	0.448–2.429	0.923
*FTO* rs9939609 (AT)	1.933	0.971–3.849	0.061	0.588	0.289–1.194	0.142
Reference TT						
*LEPR* rs1137101 (AA)	0.930	0.379–2.283	0.875	1.117	0.471–2.650	0.801
*LEPR* rs1137101 (AG)	1.227	0.530–2.838	0.633	0.924	0.407–2.097	0.850
Reference GG						
Model 2						
*FTO* rs9939609 (AA)	2.808	0.932–5.718	0.071	0.837	0.325–2.154	0.712
*FTO* rs9939609 (AT)	1.799	0.880–3.679	0.107	0.602	0.272–1.331	0.210
Reference TT						
*LEPR* rs1137101 (AA)	0.907	0.354–2.328	0.840	0.979	0.376–2.548	0.966
*LEPR* rs1137101 (AG)	1.166	0.484–2.810	0.732	0.871	0.353–2.144	0.763
Reference GG						
High protein intake	2.595	1.298–5.595	0.007	0.334	0.178–0.626	0.001
High lipid intake	2.359	0.972–5.723	0.058	0.695	0.295–1.638	0.406
High carbohydrate intake	2.333	1.117–4.873	0.024	1.457	0.670–3.172	0.342
Model 3						
*FTO* rs9939609 (AA)	4.422	1.587–12.322	0.004	0.731	0.278–1.921	0.525
*FTO* rs9939609 (AT)	1.861	0.821–4.218	0.137	0.587	0.263–1.312	0.195
Reference TT						
*LEPR* rs1137101 (AA)	0.970	0.335–2.803	0.955	1.011	0.385–2.654	0.983
*LEPR* rs1137101 (AG)	1.244	0.471–3.289	0.659	0.838	0.337–2.084	0.704
Reference GG						
High protein intake	2.531	1.160–5.524	0.002	0.3659	0.190–0.701	0.003
High lipid intake	3.123	1.127–8.653	0.028	0.726	0.300–1.756	0.477
High carbohydrate intake	2.022	0.883–4.633	0.096	1.291	0.580–2.875	0.532
Low legume intake	2.620	1.230–5.582	0.013	0.800	0.416–1.538	0.503
BMI overweight/obesity	8.951	3.941–20.328	0.000	0.311	0.133–0.726	0.007
Model 4						
*FTO* rs9939609 (AA)	4.350	1.548–12.226	0.005	0.731	0.278–1.925	0.526
*FTO* rs9939609 (AT)	1.875	0.825–4.261	0.133	0.590	0.263–1.322	0.200
Reference TT						
*LEPR* rs1137101 (AA)	0.966	0.333–2.800	0.949	1.006	0.383–2.640	0.991
*LEPR* rs1137101 (AG)	1.261	0.470–3.383	0.645	0.819	0.327–2.052	0.670
Reference GG						
High protein intake	2.549	1.154–5.633	0.003	0.369	0.191–0.713	0.003
High lipid intake	3.176	1.140–8.849	0.027	0.731	0.302–1.771	0.487
High carbohydrate intake	2.028	0.876–4.695	0.099	1.271	0.570–2.830	0.558
Low legume intake	2.649	1.240–5.659	0.012	0.957	0.425–2.155	0.916
Pre-pregnancy BMI overweight/obesity	8.951	3.932–20.373	0.000	0.311	0.133–0.726	0.011

Binary logistic regression models: Model 1 (Crude) is an unadjusted model. Model 2 is adjusted for nutrient intake (carbohydrates, lipids, proteins, legumes). Model 3 is adjusted for variables from Model 2 and pre-gestational body mass index (BMI). Model 4 is adjusted for variables from Model 3, gestational age, socioeconomic status, family type, marital status, and schooling. Abbreviations: BMI: Body Mass Index; GWG: Gestational Weight Gain; OR: Odds Ratio.

**Table 5:** Logistic regression of low birth weight (<2500 g) in relation to *FTO* rs9939609 and *LEPR* rs1137101 genetic variants (maternal and offspring), adjusted for nutritional, anthropometric, and sociodemographic factors.

Variable	Maternal Variants			Offspring Variants		
	OR	IC 95%	*p*	OR	IC 95%	*p*
Model 1						
*FTO* rs9939609 (AA)	0.588	0.185–1.868	0.368	1.842	0.508–6.685	0.353
*FTO* rs9939609 (AT)	0.450	0.173–1.173	0.103	1.344	0.565–3.197	0.503
Reference TT						
*LEPR* rs1137101 (AA)	1.833	0.541–6.205	0.330	2.274	0.793–6.522	0.126
*LEPR* rs1137101 (AG)	1.755	0.545–5.649	0.346	1.390	0.518–3.733	0.513
Reference GG						
Model 2						
*FTO* rs9939609 (AA)	0.566	0.178–1.980	0.354	1.945	0.468–8.083	0.360
*FTO* rs9939609 (AT)	0.476	0.175–1.259	0.146	1.414	0.579–3.453	0.447
Reference TT						
*LEPR* rs1137101 (AA)	2.124	0.584–7.290	0.245	2.301	0.772–6.860	0.135
*LEPR* rs1137101 (AG)	2.051	0.655–7.398	0.252	0.552	0.240–1.269	0.162
Reference GG						
High protein intake	0.697	0.298–1.436	0.365	1.273	0.457–3.545	0.645
High lipid intake	0.266	0.099–0.715	0.009	0.372	0.125–1.105	0.075
High carbohydrate intake	0.215	0.071–0.655	0.007	0.223	0.068–0.729	0.013
Model 3						
*FTO* rs9939609 (AA)	0.594	0.176–2.007	0.402	1.896	0.443–8.114	0.388
*FTO* rs9939609 (AT)	0.479	0.176–1.304	0.150	1.402	0.574–3.426	0.459
Reference TT						
*LEPR* rs1137101 (AA)	2.086	0.586–7.431	0.257	2.396	0.786–7.305	0.124
*LEPR* rs1137101 (AG)	1.983	0.578–6.802	0.276	1.210	0.424–3.455	0.721
Reference GG						
High protein intake	0.675	0.304–1.499	0.334	0.553	0.235–1.300	0.174
High lipid intake	0.267	0.099–0.719	0.009	0.366	0.122–1.092	0.072
High carbohydrate intake	0.211	0.069–0.648	0.007	0.219	0.067–0.723	0.013
Low legume intake	0.973	0.452–2.095	0.945	0.827	0.368–1.860	0.646
BMI overweight/obesity	1.104	0.445–2.740	0.831	1.182	0.450–3.102	0.735
Model 4						
*FTO* rs9939609 (AA)	0.627	0.184–2.142	0.457	1.948	0.442–8.578	0.378
*FTO* rs9939609 (AT)	0.455	0.167–1.243	0.125	1.371	0.551–3.415	0.497
Reference TT						
*LEPR* rs1137101 (AA)	2.115	0.578–7.736	0.258	2.361	0.756–7.375	0.139
*LEPR* rs1137101 (AG)	1.894	0.537–6.684	0.321	1.110	0.380–3.243	0.849
Reference GG						
High protein intake	0.660	0.293–1.490	0.318	0.551	0.232–1.313	0.179
High lipid intake	0.258	0.095–0.700	0.008	0.351	0.117–1.056	0.062
High carbohydrate intake	0.210	0.068–0.647	0.007	0.220	0.066–0.735	0.014
Low legume intake	0.961	0.444–2.078	0.919	0.834	0.369–1.887	0.663
BMI overweight/obesity	1.132	0.450–2.848	0.792	1.188	0.447–3.157	0.730

Binary logistic regression was used to analyze the association of low birth weight (<2500 g) with *FTO* rs9939609 and *LEPR* rs1137101 genetic variants (maternal and offspring), adjusted for nutritional, anthropometric, and sociodemographic factors. The models were structured as follows: Model 1 (Crude) was unadjusted; Model 2 was adjusted for nutrient intake (carbohydrates, lipids, proteins, and legumes); Model 3 was adjusted for variables from Model 2 and pre-gestational body mass index (BMI); and Model 4 was adjusted for variables from Model 3, gestational age, socioeconomic status, family type, marital status, and schooling. Abbreviations: BMI: Body Mass Index; OR: Odds Ratio.

## Data Availability

The data supporting the reported results are contained within this article and its Supplementary Material. Raw data are not publicly available due to ethical restrictions concerning patient privacy and confidentiality. However, the data may be made available upon reasonable request to the corresponding author, subject to ethical review and approval.

## Supplementary Materials

The following supporting information can be downloaded at https://www.mdpi.com/article/10.3390/ijms27083413/s1.

## Abbreviations

The following abbreviations are used in this manuscript:

ALFA	Allele Frequency Aggregator
BMI	Body Mass Index
FTO	Fat mass and obesity-associated gene
GWAS	Genome-Wide Association Study
GWG	Gestational Weight Gain
HWE	Hardy–Weinberg Equilibrium
IOM	Institute of Medicine (USA)
LBW	Low Birth Weight
LEP	Leptin
LEPR	Leptin receptor
NCBI	National Center for Biotechnology Information
OR	Odds Ratio
PCR	Polymerase Chain Reaction
PGH	Placental Growth Hormone
SNPs	Single Nucleotide Polymorphisms
SNV	Single Nucleotide Variant
