# Supplementary material for: Role of FTO rs9939609 and LEPR rs1137101 Genetic Variants in Gestational Weight Gain and Neonatal Weight Among Pregnant Adolescents
Source: Int J Mol Sci. 2026 Apr 10;27(8):3413. doi: 10.3390/ijms27083413 (PMC13117026; doi:10.3390/ijms27083413)
Supplement: Supplementary file 1 [file ijms-27-03413-s001.zip › ijms-4212014-supplementary.pdf]

Supplementary material

Supplementary Table S1. Primer sequences for genotyping the *FTO* rs9939609 and *LEPR* rs1137101 variants.

**Variants**	**Sequences**
FTO fw	5′ CAGTTATGCATTTAGAATGTCTG 3′
FTO rv	5′ GCTCTCCCACTCCATTTCTGA 3′
LEPR fw	5′ AACAGCCAAACTCAACGACAC 3′
LEPR rv	5′ TTTAGACCTATTATCATCATTT 3′

Supplementary table S2. Distribution of *FTO* rs9939609 and *LEPR* rs1137101 genetic variants among mothers and their offspring.

FTO rs9939609 in the mother. *n* = 353					
Genotype	Frequencies	Ratio	Allele A	Allele T	*n* alleles
AA	50	0.142	100	0	100
AT	87	0.246	87	87	174
TT	216	0.612	0	432	432
Total	353	1	187 (0.264)	519 (0.735)	706
Not in Hardy-Weinberg equilibrium, *p* = <0.0001					
FTO rs9939609 in their offspring, n= 305					
AA	42	0.138	84	0	84
AT	77	0.252	77	77	154
TT	186	0.610	0	372	372
Total	305	1	161 (0.263)	449 (0.736)	610
Not in Hardy-Weinberg equilibrium, *p* = <0.001					
LEPR rs1137101 in the mother. *n* = 355					
Genotype	Frequencies	Ratio	Allele A	Allele G	*n* alleles
AA	106	0.299	212	0	212
AG	179	0.504	179	179	358
GG	70	0.197	0	140	140
Total	355	1	391 (0.55070)	319 (0.44929)	710
In Hardy-Weinberg equilibrium, *p* = 0.720					
LEPR rs1137101 in their offspring, *n* = 309					
AA	83	0.269	166	0	166
AG	141	0.456	141	141	282
GG	85	0.275	0	170	170
Total	309	1	307 (0.49676)	311 (0.50323)	618
In Hardy-Weinberg equilibrium, *p* = 0.120					

Table supplementary S3. Distribution (%) of food and nutrients intake according to genotype variants of *FTO* rs 9939609 and *LEPR* rs 1137101.

Maternal genetic variants	*FTO* rs9939609*				*LEPR* rs1137101**			
	AA n=50	AT n=87	TT n=216	*p*	GG n=106	GA n=179	AA n=70	*p*
Inadequate energy	68	88	81	0.089	84	79	83	0.762
Inadequate fiber	100	98	100	0.235	100	99	100	0.605
Inadequate water	89	69	70	0.094	73	70	75	0.808
Inadequate protein	68	63	62	0.853	60	62	62	0.961
Inadequate lipids	71	84	81	0.382	76	80	83	0.417
Inadequate carbohydrates	61	69	73	0.418	70	68	74	0.666
Excessive cereals	82	73	78	0.583	84	79	71	0.135
Low fruits and vegetables	82	71	69	0.377	97	97	94	0.501
Low legumes	57	27	48	0.014	68	56	46	0.028
Excessive fats	61	71	61	0.487	78	85	79	0.539
Diet characteristics								
Insufficient diet	39	41	45	0.564	35	48	41	0.367
Unbalanced diet	57	37	54	0.102	38	46	57	0.053
Unvaried diet	71	45	48	0.057	37	50	60	0.023
Incomplete diet	96	88	90	0.477	89	90	91	0.979
Unsafe diet	71	45	47	0.052	37	49	60	0.021

*n = 353; ** n = 355

Fiber intake had a median of 15 g; meanwhile water consumption was 1026 mL without difference by genetic variants from *FTO* rs9939609 and *LEPR* rs1137101, *p* by Kruskal-Wallis.
